# Approximating the double-cut-and-join distance between unsigned genomes

**DOI:** 10.1186/1471-2105-12-S9-S17

**Published:** 2011-10-05

**Authors:** Xin Chen, Ruimin Sun, Jiadong Yu

**Affiliations:** 1 Division of Mathematical Sciences, School of Physical and Mathematical Sciences, Nanyang Technological University, Singapore

## Abstract

In this paper we study the problem of sorting unsigned genomes by double-cut-and-join operations, where genomes allow a mix of linear and circular chromosomes to be present. First, we formulate an equivalent optimization problem, called maximum cycle/path decomposition, which is aimed at finding a largest collection of edge-disjoint cycles/AA-paths/AB-paths in a breakpoint graph. Then, we show that the problem of finding a largest collection of edge-disjoint cycles/AA-paths/AB-paths of length no more than *l* can be reduced to the well-known degree-bounded *k*-set packing problem with *k* = 2*l.* Finally, a polynomial-time approximation algorithm for the problem of sorting unsigned genomes by double-cut-and-join operations is devised, which achieves the approximation ratio  for any positive ε. For the restricted variation where each genome contains only one linear chromosome, the approximation ratio can be further improved to

## Introduction

A fundamental problem in the study of genome rearrangements is to compute the genomic distance between two genomes based on their gene orders, where the genomic distance is generally defined as the minimum number of evolutionary operations necessary to transform one genome to another. This problem has been extensively studied in the last two decades [1-4].

The choices of genome evolutionary operations include reversals (also called inversions), translocations, fissions, fusions, transpositions and block-interchanges. To unify all these classical operations, Yancopoulos et al [4] introduced a single operation, called *double-cut-and-join* (DCJ). It basically cuts a genome in two places and then joins the resulting four ends in a new way. Noticeably, computing the genomic distance based on DCJ operations can be applied between two genomes that allow a mix of linear and circular chromosomes to be present.

The complexity of computing the genomic distance between two genomes seems to largely depend on the availability of gene strand information rather than on the choice of evolutionary operations. For instance, the problem of sorting by reversals is tractable when gene strand information is available [5,6], but becomes intractable once gene strand information is not available [7]. The same conclusion also applies to the problem of sorting genomes by the double-cut-and-join operations. The DCJ operation was initially introduced to sort two *signed* genomes in [4,8], where a simple formula was derived to compute the genomic distance in linear time. It was recently used to sort two *unsigned* genomes in [9,10]; in this case, one instead has to tackle an NP-hard optimization problem.

To tackle an NP-hard optimization problem, it is of highly practical interest to develop a polynomial-time approximation algorithm with provable performance guarantee. For the problem of sorting by double-cut-and-join operations, in the case of unsigned *uni-chromosome* genomes, Chen presented in [9] an approximation algorithm with a performance ratio of  for any positive ε. In the case of unsigned *linear* genomes, Jiang et al [10] recently devised a 1.5-approximation algorithm.

In this paper we study the problem of sorting unsigned genomes by double-cut-and-join operations in the more general case where genomes allow a mix of *linear* and *circular* chromosomes to be present. The main goal is to devise a polynomial-time approximation algorithm for this NP-hard problem. To this end, we first formulate a new and equivalent combinatorial optimization problem, called *maximum cycle/path decomposition*, which is aimed at finding a largest collection of edge-disjoint cycles/AA-paths/AB-paths in the breakpoint graph constructed from two input genomes. Then, we show that the problem of finding a largest collection of edge-disjoint cycles/AA-paths/AB-paths of length no more than *l* in the breakpoint graph can be reduced to the well-known *degree-bounded k-set packing problem*, where *k* = *2l.* Finally, we present a polynomial-time approximation algorithm for the problem of sorting unsigned genomes by DCJ operations and then obtain its approximation ratio  for any positive ε. To our best knowledge, it is the first polynomial-time approximation algorithm of this kind.

## Methods

### Preliminaries

#### Genes, chromosomes, and genomes

A *gene* is a stretch of DNA with two ends: the 3′ end and the 5′ end. Both are called the *extremities* of a gene. A *chromosome* is a single double-stranded DNA molecule that contains a sequence of *genes.* It can be either a linear molecule or a circular molecule. For a linear chromosome, there is a *telomere* marker located at each of its two ends. A *genome* is the whole collection of chromosomes in a cell. For example, the following gives a genome containing two linear chromosomes and one circular chromosome.

Genome A = {(o, *a*, c, *d*, *o*), (*b*, *e*), (*o*, *f*, *g*, *o*)}

Here we use the symbol ‘o’ to represent a telomere marker, further indicating that the corresponding chromosome is linear. An *unsigned* alphabetical symbol is used to represent a gene for which the 3′ end and 5′ end are not yet identified. Accordingly, a genome in this representation is called *unsigned.* On the other hand, if every gene is represented as a *signed* symbol where the sign indicates which extremity is the 3′ end (and the other extremity must be the 5′ end), then the corresponding genome is called *signed.* For a signed genome, Bergeron et al [8] introduced a new and equivalent representation, which is a set of *adjacencies* between extremities from different genes or between telomeres and extremities. For example, we may represent a signed genome

as

where *a_h_* and *a_t_* represents the 5′ end and 3′ ends of gene *a*, respectively.

#### Sorting by double-cut-and-joins

The *double-cut-and-join* (DCJ) is an operation that cuts a genome in two places and joins the resulting four ends in a new way. Specifically, the cuts are applied between two adjacent extremities from different genes, between a telomere and its adjacent gene extremity, or between the two extremities of a null chromosome if necessary. The DCJ operation was first introduced by Yancopoulos et al [4] and later refined by Bergeron et al [8] to unify all the classical genome rearrangement events including inversions, translocations, fissions, fusions, transpositions, block-interchanges, circularization and linearization.

Given two unsigned genomes *A* and *B* on the same set of genes, the problem of *sorting unsigned genomes by DCJ operations* (UDCJ) is defined to find a shortest sequence of DCJ operations that transform one genome into the other. The length of such a sequence is called the *double-cut-and-join distance* between two genomes *A* and *B*, and denoted by *d_D_*_CJ_(*A*, *B*)*.*

**Example 1. ***Let two unsigned genomes be*

*Genome A has two linear chromosomes and one circular chromosome*, *while genome B has two linear chromosomes and two circular chromosomes. Sorting A into B can*, *for example*, *be done in the following four DCJ operations*, *where the places to be cut are underlined:*

*We will see later that at least four DCJ operations are needed to transform one genome into the other. Therefore*, *the DCJ distance between A and B is d_DCJ_*(*A*, *B*) = 4.

#### The degree-bounded k-set packing problem

Given a base set *S* and a collection *S* of subsets of *S*, the *set packing* problem asks for the maximum number of pairwise disjoint subsets in *S.* The *k-set packing* problem is a restricted variant of the set packing problem where every subset in *S* has size at most *k.* If in addition the number of occurrences in *S* of any element is upper bounded by a constant Δ, then it reduces to the *degree-bounded k-set packing* problem. The following theorem states the best-to-date approximability and its detailed proof can be found in [11].

**Theorem 2** ([12]). *The degree-bounded k-set packing problem can be approximated within ratio**in polynomial time*, *for any positive ε.*

This algorithm is denoted by APPROX-SP(*k*, Δ) and will be used in our approximation for computing the DCJ distance between two unsigned genomes. Its running time complexity is  as shown in [11].

### Basic facts

#### The breakpoint graph

The breakpoint graph (also called edge graph or comparison graph) is first introduced in [1] and widely used to compute the genomic rearrangement distances. Let *A* and *B* be two unsigned genomes defined on the same set of *n* genes containing*;l_A_* and *l_B_* linear chromosomes, respectively. We construct the *breakpoint graph G*(*A*, *B*) = (*V*, *E* = *E_b_* ∪ *E_g_*) as follows. Each vertex in |*V|* corresponds to a distinct gene or a telomere, so |*V*| = *n* + 2*l_A_* + 2*l_B_*. Every adjacency in *A* forms a *black* edge belonging to *E_b_* and every adjacency in *B* forms a *gray* edge belonging to *E_g_.* It is easy to see that |*E_b_*| = *n* + *l_A_*, *|E_g_|* = *n* + *I_B_*, and |*E*| = 2*n* + *l_A_* + *l_B_.* Moreover, every telomere of genome *A* (resp. genome *B*) has degree one and is incident to a black (resp. gray) edge, whereas every gene has degree four and is incident to two black and two gray edges. For short, a telomere of genome *A* is referred to as an A-telomere, and a telomere of genome *B* as a B-telomere. Note that the number of A-telomeres is not necessarily equal to the number of B-telomeres in a breakpoint graph.

**Figure 1:**
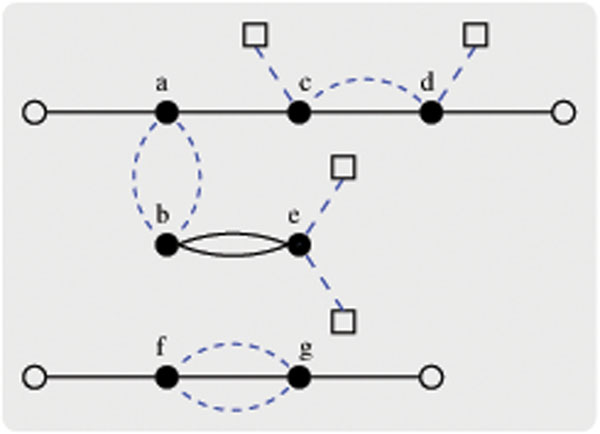
**A breakpoint graph between two genomes.** The input two genomes are *A* = {(o, *a*, *c*, *d*, *o*), (*b*, *e*), (*o*, *f*, *g*, *o*)} and *B* = {(*a*, *b*), (*o*, *c*, *d*, *o*), (*o*, *e*, *o*), (*f*, *g*)}*.* The solid dots, hollow circles and squares are vertices representing genes, the telomere markers of genome *A* and of genome *B*, respectively. The solid and dashed lines are used to represent black and gray edges, respectively.

**Example 3. ***Consider the two unsigned genomes A and B given in Example 1*, *where l_A_* = 2 *and l_B_* = 2. *The breakpoint graph G*(*A*, *B*) = (*V*, *E* = *E_b_* ∪ *E_g_*) *is depicted in Figure*1, *in which |V|* = 15**,** |*E_b_*| = 9, *|E_g_|* = 9, *and |E|* = 18.

#### The cycle/path decomposition

A cycle/path in the breakpoint graph *G*(*A*, *B*) is called *alternating* if its edges are alternatively black and gray. From now on, whenever we mention cycles/paths in a breakpoint graph, they are alternating and edge-disjoint. A path is called an *AA-path* (resp. *BB-path*) if it connects two A-telomeres (resp. two B-telomeres) or an *AB-path* if it connects an A-telomere and a B-telomere. The *length* of a cycle/path is referred to as the number of black edges that it contains.

**Figure 2:**
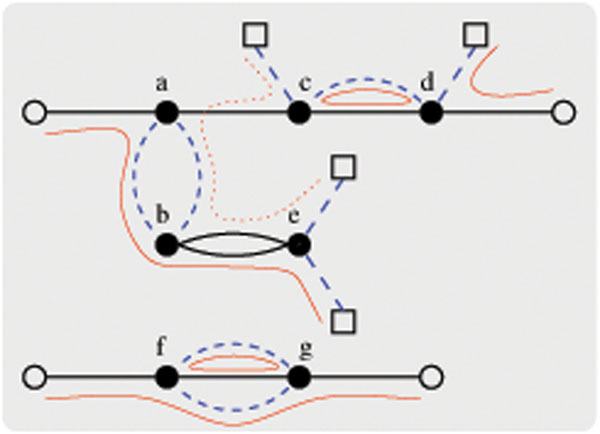
**A maximum cycle/path decomposition of the breakpoint graph depicted in Figure**1. It consists of two cycles, one AA-path, two AB-paths and one BB-path. Hence, we obtain *c* = 2, *I_AA_* = 1 and *I_AB_* = 2 so that *d_DCJ_* = 4.

Since every telomere vertex has degree one and every gene vertex has two incident black edges and two incident gray edges, there always exists a *cycle/path decomposition* of *G*(*A*, *B*) into edge-disjoint cycles, AA-paths, AB-paths and BB-paths. A *maximum cycle/path decomposition* refers to a cycle/path decomposition that contains the maximum number of edge-disjoint cycles, AA-paths and AB-paths. Note that this maximum number does not take into account any BB-paths. The *maximum cycle/path decomposition* problem is hence defined as the problem of finding such a maximum cycle/path decomposition of a breakpoint graph. See Figure 2 for an example.

#### Sorting signed genomes

Before proceeding to study the problem of sorting unsigned genomes by DCJ operations, we take a first look at the case of signed genomes. Note that the above concept of breakpoint graph extends naturally to two signed genomes  and  It can be done by replacing each signed gene by its two (unsigned) extremities in the relative order. Yancopoulos, et al. [4] further proposed to close each AA-path into a cycle by adding a gray edge connecting two A-telomeres, close each BB-path into a cycle by adding a black edge connecting two B-telomeres, and close each AB-path into a cycle by identifying the A-telomere and B-telomere (with the B-telomere eliminated). As is customary, no edge is drawn between two extremities of the same gene in the breakpoint graph.

Given a breakpoint graph for two signed genomes  and  we note that its cycle/path decomposition is unique and trivial because every vertex in  has degree at most two. The following theorem implies that computing the DCJ distance between two signed genomes can be done in linear time.

**Theorem 4** ([4]). *Let**and**be two genomes defined on the same set of n genes*, *then we have*

*where**is the number of black edges and**the number of cycles in the breakpoint graph**after closing all the paths into cycles.*

#### Sorting unsigned genomes

Theorem 7 that we will present below establishes the connection between the cycle/path decomposition of a breakpoint graph and the DCJ distance between two unsigned genomes. Its proof uses the following two lemmas (i.e., Lemmas 5 and 6).

**Lemma 5. ***For every cycle/path decomposition of G*(*A*, *B*), *there exists a signed version**and**of genomes A and B such that*

*where b is the number of black edges in the breakpoint graph G*(*A*, *B*) *and c* (*resp. I_AA_ and I_AB_*) *is the number of cycles* (*resp. AA-paths and AB-paths*) *in the given cycle/path decomposition of G*(*A*, *B*)*.*

*Proof.* Note that every gene vertex would be visited twice if we traverse all the cycles/paths in a fixed cycle/path decomposition of *G*(*A*, *B*)*.* When a gene vertex (e.g., gene *a*) is visited for the first time, we may assume that we are visiting the 5′ end of the gene (denoted as *a_h_*). When it is visited for the second time, we may assume that we are visiting the 3′ end of the gene (denoted as *a_t_*). To obtain a signed genome  (represented as a set of adjacencies), we form an adjacency for every two extremities (or one extremity and one A-telomere) that are connected by a black edge in the given cycle/path decomposition. Similarly, to obtain a signed genome  we form an adjacency for every two extremities (or one extremity and one B-telomere) that are connected by a gray edge in the given cycle/path decomposition. It is easy to see that the resulting genomes  and  are the signed version of genomes *A* and *B*, respectively.

Moreover, the breakpoint graph  before closing its paths into cycles, preserves all the cycles/paths from the given cycle/path decomposition of *G*(*A*, *B*)—that is, there are still *b* black edges, *I_AA_* AA-paths, *I_AB_* AB-paths and *I_BB_* BB-paths. After closing paths into cycles, the breakpoint graph  would have  black edges (as we close each BB-path into a cycle by adding one black edge) and  cycles. It hence follows from Theorem 4 that

**Lemma 6.*** For every signed version**and**of genomes A and B*, *there exists a cycle/path decomposition of G*(*A*, *B*) *such that*

*where b is the number of black edges in the breakpoint graph G*(*A*, *B*) *and c* (*resp. I_AA_ and I_AB_*) *is the number of cycles* (*resp. AA-paths and AB-paths*) *in this cycle/path decomposition of G*(*A*, *B*)*.*

*Proof.* Observe that we would obtain the breakpoint graph *G*(*A*, *B*) if we combine two extremity vertices of a same gene into a single vertex in the breakpoint graph  (before closing paths into cycles).

Therefore, the trivial cycle/path decomposition of  naturally gives rise to a cycle/path decomposition of *G*(*A*, *B*) which preserves the same numbers of black edges/cycles/AA-paths/AB-paths/BB-paths (denoted as *b*, *c*, *I_AA_*, *I_AB_* and *I_BB_*, respectively). After closing paths into cycles, the breakpoint graph  would have  black edges and  cycles, as justified in the preceding lemma. By Theorem 4, we then have 

**Theorem 7. ***Let A and B be two unsigned genomes defined on the same set of genes. Then*, *we have*

*d_DCJ_*(*A*, *B*) = *b –* (*c* + *I_AA_* + *I_AB_*)

*where b is the number of black edges in G*(*A*, *B*) *and c* (*resp.*, *I_AA_ and I_AB_*) *is the number of cycles* (*resp.*, *AA-paths and AB-paths*) *in a maximum cycle/path decomposition of G*(*A*, *B*)*.*

*Proof.* Let us consider a maximum cycle/path decomposition of *G*(*A*, *B*)*.* By Lemma 5, we would obtain a signed version  and  of genomes *A* and *B* such that  It means that genome  can be transformed into genome  with a sequence of  DCJ operations. Observe that this same sequence of DCJ operations can be also used to transform genome *A* into genome *B.* It hence follows that 

Assume now that a sequence of *d_DCJ_*(*A*, *B*) DCJ operations can be applied to transform genome *A* into genome *B.* Let  be a signed version of genome *A* in which all genes are positive. We then apply the same sequence of *d_DCJ_*(*A*, *B*) DCJ operations to the signed genome  The resulting genome  would be genome *B* if we disregard all the gene signs; in other words,  shall be a signed version of genome *B.* Thus,  On the other hand, by Lemma 6, there exists a cycle/path decomposition of *G*(*A*, *B*) such that  Thus, *d_DCJ_*(*A*, *B*) ≥ *b –* (c + *I_AA_* + *I_AB_*)*.*

## Results

### The approximation algorithm

Note that, for a given pair of genomes, the number of black edges is fixed without regard to any cycle/path decomposition. Theorem 7 hence suggests a way to approximate the DCJ distance between two unsigned genomes via maximizing the number of cycles/AA-paths/AB-paths in a cycle/decomposition of the breakpoint graph. To do so, our proposed approximation algorithm performs three subroutines to find edge-disjoint cycles/paths of length one, of length two, and of length no more than three, respectively.

#### Finding edge-disjoint cycles/paths of length one

**Lemma 8.*** There exists a maximum cycle/path decomposition of G*(*A*, *B*) *which contains a largest collection of edge-disjoint cycles/AA-paths/AB-paths of length one.*

**Figure 3:**
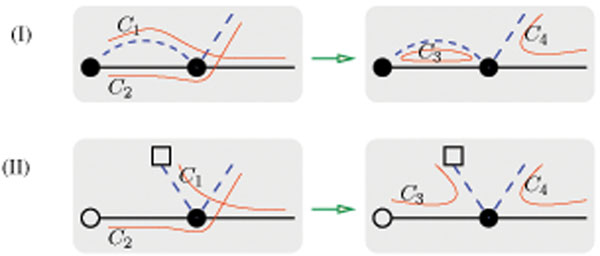
**Two possible cases of the cycles/paths *C*_3_ of length one.** (I) a cycle, and (II) an AB-path.

*Proof.* Let *C* be a maximum cycle/path decomposition of *G*(*A*, *B*) and *C*_1_ a largest collection of edge-disjoint cycles/AA-paths/AB-paths of length one in *G*(*A*, *B*)*.* If *C*_3_ is a cycle/AA-path/AB-path contained in *C*_1_ but not in *C* (please refer to Figure 3), then the maximum cycle/path decomposition *C* shall contain the cycles/paths *C*_1_ and *C*_2_ (note that they are not necessarily distinct). In this case, we would modify the maximum cycle/path decomposition *C* as follows: take one gene vertex of the only black edge of *C*_3_ and re-connect its incident black edges and gray edges in a new way. Consequently, *C*_1_ and *C*_2_ would be replaced by the two distinct cycles/paths *C*_3_ and *C*_4_ in *C.* Suppose by contradiction that the above modification decreases the number of cycles/AA-paths/AB-paths so that the new *C* is no longer a maximum cycle/path decomposition. Note first that this would happen only when *C*_1_ and *C*_2_ are two distinct cycles/AA-paths/AB-paths but *C*_4_ is a BB-path. Since no AA-path in *G*(*A*, *B*) could be of length one, *C*_3_ is either a cycle or an AB-path. Hence, *C*_3_ and *C*_4_ together use at least two B-telomeres and at most one A-telomere, and so do *C*_1_ and *C*_2_ together. Since neither *C*_1_ nor *C*_2_ is a BB-path, they together shall use A-telomeres no less than B-telomeres, contrasting the previously established fact that they together use at least two B-telomeres and at most one A-telomere. Continue this process with the remaining cycles/AA-paths/AB-paths that are contained in *C*_1_ but not in *C.* It would necessarily end up with a maximum cycle/path decomposition that contains a largest collection of edge-disjoint cycles/AA-paths/AB-paths of length one.

It is worth noticing that there might be no maximum cycle/path decomposition of *G*(*A*, *B*) which could contain all the cycles/AA-paths/AB-paths of length one.

**Lemma 9.*** The problem of finding a largest collection of edge-disjoint cycles*, *AA-paths and AB-paths of length one in the breakpoint graph G*(*A*, *B*) *is solvable in polynomial time.*

*Proof.* We can transform a problem instance of finding a largest collection of edge-disjoint cycles/AA-paths/AB-paths of length one into an instance of the 2-set packing problem, where the base set *S* contains all the edges of *G*(*A*, *B*) and each subset of the collection *S* is comprised of edges of a cycle/AA-path/AB-path of length one in *G*(*A*, *B*)*.* The 2-set packing problem can be reduced to the maximal matching problem which is well-known to be solvable in polynomial time by a simple greedy algorithm [13].

Following Lemma 9, we assume from now on that there does not exist any cycle/AA-path/AB-path of length one in the breakpoint graph *G*(*A*, *B*)*.*

#### Finding edge-disjoint cycles/paths of length two or three

The following lemma gives an upper bound on the number of distinct cycles/paths of length less than or equal to *l* that traverse a common edge. Although this upper bound is no way the tight one, it is already enough for our purpose to devise an approximation algorithm.

**Lemma 10.*** Every edge in the breakpoint graph G*(*A*, *B*) *belongs to at most* (2^2^*^l^*^+1^ × *l^2^*) *distinct cycles/AA-paths/AB-paths of length less than or equal to l.*

*Proof.* Let us consider a breadth-first traversal of edges in the breakpoint graph *G*(*A*, *B*) that starts at a given edge *e*, and then count all the cycles/AA-paths/AB-paths of length less than or equal to *l* that use the edge *e*. Note that every vertex is incident to at most two black edges and at most two gray edges and also that every edge is at distance at most (*2l –* 1) from another edge of the same cyle/path of length less than or equal to *l*. The traversal of every such cycle/path will end at two edges at distance *i* and *j* from the root edge *e*, respectively, such that *i* ,*j* ≥ 0 and *i* + *j* ≤ *2l –* 1. If we fix values for *i* and *j*, there are at most 2*^i^* × 2*^j^* = 2*^i^*^+^*^j^* ≤ 2^2^*^l–^*^1^ cycles/paths whose traversals end at two edges in distance *i* and *j* from the root edge *e*, respectively. For all the combinations of values *i* and *j* such that *i* ≤ 2*l –* 1 and *j* ≤ 2*l –* 1, there are at most *2l* × *2l* × 2^2^*^l–^*^1^ = 2^2^*^l^*^+1^ × l*^2^* cycles/paths to be reached; in other words, there are at most 2^2^*^l^*^+1^ × l^2^ cycles/paths of length less than or equal to *l* that use the edge *e*.

To find a largest collection of edge-disjoint cycles/paths of length no more than *l*, we may construct a collection *S* of subsets of the base set *S*, where *S* is comprised of all the edges in *G*(*A*, *B*) and each subset of *S* is comprised of edges of a distinct cycle/AA-path/AB-path of length no more than *l* in *G*(*A*, *B*)*.* Then, we obtain an instance (*S*, *S*) of the *k*-set packing problem where *k* = 2*l.* Further by the above lemma, we obtain the following observations.

**Corollary 11. ***Finding a largest collection of edge-disjoint cycles/AA-paths/AB-paths of length two can he transformed into an instance of the degree-bounded 4-set packing problem.*

**Corollary 12. ***Finding a largest collection of edge-disjoint cycles/AA-paths/AB-paths of length no more than three can he transformed into an instance of the degree-bounded 6-set packing problem.*

It is worth noting that, if, as previously done in [10] on the breakpoint graph, all the paths are closed into cycles to make a maximum cycle decomposition instead of a maximum cycle/path decomposition, we would not know whether the above corollaries (and hence our approximation algorithm proposed later) are still valid. Two lemmas below further follow from Theorem 2.

**Lemma 13. ***The problem of finding a largest collection of edge-disjoint cycles/AA-paths/AB-paths of length two in the breakpoint graph G*(*A*, *B*) *can be approximated with ratio**in polynomial time*, *for any positive ε.*

**Lemma 14.*** The problem of finding a largest collection of edge-disjoint cycles/AA-paths/AB-paths of length no more than three in the breakpoint graph G*(*A*, *B*) *can be approximated with ratio**in polynomial time*, *for any positive ε.*

#### Algorithm details

Let *A* and *B* be two unsigned genomes defined on the same set of genes. Given a breakpoint graph *G*(*A*,*B*), our proposed algorithm for the cycle/path decomposition is summarized below:

1. Find a largest collection *C*_1_ of cycles/AA-paths/AB-paths of length one by a greedy algorithm;

2. Remove from the breakpoint graph all the edges used by the cycles/AA-paths/AB-paths of *C*_1_*;*

3. Find a collection *C*_2_ of cycles/AA-paths/AB-paths of length two by Algorithm APPROX-SP(*k*, Δ);

4. Find a collection *C*_3_ of cycles/AA-paths/AB-paths of length no more than three by Algorithm APPROX-SP(*k*, Δ);

5. Decompose the remaining edges arbitrarily into a collection *C*_4_ of cycles/AA-paths/AB-paths/BB-paths;

6. Output either *C*_1_ ∪ *C*_2_ ∪ *C*_4_ or *C*_1_ ∪ *C*_3_ ∪ *C*_4_, depending on which one has the larger size.

For a maximum cycle/path decomposition, let *r*_2_ denote the number of cycles/AA-paths/AB-paths of length two, *r*_3_ the number of cycles/AA-paths/AB-paths of length three, and *r*′ the total number of cycles/AA-paths/AB-paths. Let *r* be the total number of cycles/AA-paths/AB-paths in the cycle/path decomposition returned by our proposed algorithm. The cycles/AA-paths/AB-paths of length one are all excluded from the above counts since it would not affect the worst-case algorithmic performance. We find the worst-case approximation ratio of our algorithm by solving the following optimization problem:

The second constraint is due to the fact that every cycle/AA-path/AB-path of length four or larger uses at least four black edges. The third and fourth constraints follow from Lemmas 13 and 14, respectively. By solving the above *fractional linear programming* problem (please refer to Lemma 2.3 of [14]), we would obtain the maximum objective function value being  which indeed gives the performance ratio of our proposed algorithm.

**Theorem 15.*** The problem of sorting unsigned genomes by DCJ operations can be approximated within ratio**in polynomial time*, *for any positive ε.*

#### Sorting unsigned permutations

A genome can be represented as an unsigned permutation when it contains only one linear chromosome. For this restricted case, we can improve the approximation ratio further by applying the following result from [14] in place of Lemma 13.

**Lemma 16** ([14]). *Let A and B be two unsigned uni-chromosome genomes. The problem of finding a largest collection of edge-disjoint cycles/AA-paths/AB-paths of length two in the breakpoint graph G*(*A*, *B*) *can be approximated with ratio**in polynomial time*, *for any positive ε.*

In view of this lemma, the third constraint in the above fractional linear programming problem can be replaced by the inequality  which hence leads to the following theorem.

**Theorem 17. ***The problem of sorting unsigned permutations by DCJ operations can be approximated within ratio**in polynomial time*, *for any positive ε.*

## Conclusions

Since the introduction of the NP-hard problem of sorting unsigned genomes by double-cut-and-join operations in [9], the polynomial-time approximation algorithms have been developed only under two restricted genome models. The first one is intended for sorting uni-chromosome genomes and its best-to-date performance ratio is  for any positive ε [9]. The second one is intended for sorting linear genomes and its best-to-date performance ratio is 1.5 [10]. In this paper, we have presented an approximation algorithm for the problem of sorting unsigned genomes by double-cut-and-join operations in the general case where genomes allow a mix of linear and circular chromosomes to be present. The performance ratio thus achieved is  for any positive ε. In addition, for the first restricted genome model mentioned above, an improved performance ratio of  is also achieved. However, the proposed algorithm is mainly of theoretical interest rather than the practical use, due to its huge factor polynomial running time 

Conceptually, our proposed algorithm operates in the same spirit as many previous algorithms for approximating the genomic distance via genome rearrangement operations [1,10,14,15]. However, when we began this work, it was not clear whether the problem of finding a largest collection of edge-disjoint cycles/AA-paths/AB-paths of length two or three can be reduced to a *degree-bounded k*-set packing problem (rather than a general *k*-set packing problem). In this paper we established this reduction, which then leads to the improved approximation ratios.

## Competing interests

The authors declare that they have no competing interests.

## Authors' contributions

XC conceived the study. All authors contributed to the algorithm analysis, read and approved the final manuscript.
